# Vibration and Stray Flux Signal Fusion for Corrosion Damage Detection in Rolling Bearings Using Ensemble Learning Algorithms

**DOI:** 10.3390/s26010233

**Published:** 2025-12-30

**Authors:** José Pablo Pacheco-Guerrero, Israel Zamudio-Ramírez, Larisa Dunai, Jose Alfonso Antonino-Daviu

**Affiliations:** 1 Engineering Faculty, San Juan del Río Campus, Universidad Autónoma de Querétaro, Av. Río Moctezuma 249, San Juan del Río 76807, Querétaro, Mexico; 2 Department of Graphic Engineering, Universitat Politècnica de València (UPV), Camino de Vera s/n, 46022 Valencia, Spain; 3 Instituto Tecnológico de la Energía, Universitat Politècnica de València (UPV), Camino de Vera s/n, 46022 Valencia, Spain

**Keywords:** vibration, stray flux, induction motors, corrosion, rolling bearings, ensemble learning algorithms

## Abstract

Early fault diagnosis in induction motors is important to maintain correct operation in terms of energy and efficiency, as well as to achieve a reduction in costs associated with maintenance or unexpected stoppages in production processes. These motors are widely used in industry due to their reliability, low cost, and great robustness; however, over time, they may be exposed to wear that can affect their performance, endanger the integrity of operators, or cause unexpected shutdowns that generate economic losses. Corrosion in the bearings is one of the most common failures, which is mainly triggered by high humidity in combination with high temperatures. However, despite its relevance, it has not been widely explored as a cause of failure in induction motors. Unlike failures that occur in specific or localized areas, corrosion in bearings does not manifest through specific frequencies associated with the phenomenon, since the corrosion occurs extensively on the surface of the raceway, making early diagnosis difficult with conventional techniques based on spectral analysis. Therefore, this work proposes an approach for the analysis of magnetic stray flux and vibration signals under different levels of corrosion using statistical and non-statistical parameters to capture variations in the dynamic behavior of the motors while employing genetic algorithms to select the most relevant parameters for each signal and optimize the configuration of an ensemble learning algorithm. The classification of the bearing condition is achieved using support vector machines in combination with the bagging method, which increases the robustness and accuracy of the model in the presence of signal variability. A classification accuracy between the healthy state and two gradualities greater than 99% was obtained, indicating that the proposed approach is reliable and efficient for corrosion diagnosis.

## 1. Introduction

Induction motors (IMs) are tools that have revolutionized the world due to their wide range of applications, including in industrial equipment such as pumps, compressors, and conveyor belts, as well as in sectors such as manufacturing, healthcare, and the automotive industry, among others. For this reason, they are among the most widely used types of motor worldwide [1]. They are characterized by low cost and high operational reliability, as they can continue operating even in the presence of certain faults.

Some studies, such as [2], indicate that by 2030, the growing global electricity demand will still be supplied by fossil fuels. This study mentions that 42% of the world’s energy consumption occurs in the industrial sector, with IMs being responsible for approximately 28% of that total; that is, two-thirds of the world’s energy is attributed to these machines. When an IM presents faults such as broken rotor bars or damage to bearing elements, such as the outer race or cage, friction between its components tends to increase. In turn, this generates heat and additional energy losses, reducing efficiency and causing higher electric current consumption [3], which results in higher billing costs. Given the above, these losses translate into a direct increase in operating costs, including higher expenses due to electrical energy consumption and a reduction in the equipment’s useful life, which could lead to unexpected shutdowns and higher maintenance or replacement costs. These effects not only impact the energy efficiency of industrial processes but also contribute to unsustainable energy use, reinforcing the need for early diagnosis strategies that help reduce losses and optimize the performance of induction motors. Some studies have shown that approximately 45% of faults occur in bearings, 35% in the stator, 10% in the rotor, and the remaining 10% in other components. Bearing failures, therefore, represent almost half of all motor failures [4].

It is known that bearing failures are mainly due to adverse conditions such as high temperatures in combination with humidity and high rotor vibration caused by excessive torque, among others. When these are combined with internal factors such as improper installation, deterioration of lubrication, heat, friction, and contamination, these conditions can lead to one of the most critical problems, which is corrosion due to humidity. In addition, bearing failure often produces rotor eccentricity, which in turn translates into magnetic unbalance pull, generating additional loads acting on the bearings. Additionally, this type of problem is a source of excessive vibrations due to the resulting change in stiffness, giving rise to secondary problems, such as broken rotor bars and, in severe cases, possible rotor breakage if the problem is not corrected in time [5]. It is estimated that around 10 billion bearings are manufactured annually worldwide, and a large percentage of them tend to fail, requiring the replacement of nearly 50 million bearings due to various operational problems [6]. The most common reasons for replacement are fatigue (one-third of cases), lubrication problems (one-third), contamination (one-sixth), and other causes such as improper handling, incorrect mounting, and overloads. According to [6], corrosion in induction motor bearings can be divided into three main types—corrosion due to humidity, vibrocorrosion, and contact corrosion—highlighting that these issues have the greatest impact and recurrence in real industrial environments. In addition, they occur throughout the bearing as diffuse and non-uniform areas, ultimately causing surface wear over extensive regions.

Given the above, a wide variety of fault diagnosis methodologies have been proposed, ranging from conventional frequency-based techniques, such as the fast Fourier transform (FFT), to approaches based on machine learning (ML) and artificial intelligence (AI). Some comparative studies [7,8,9,10] have shown that ML- and AI-based techniques generally provide the most accurate results, highlighting methods such as support vector machines (SVMs), Bayesian classifiers, and decision trees for their effectiveness.

In addition, other proposed approaches are based on frequency- and time–frequency-domain transforms, such as wavelet-based and FFT-based methods, as described in [11,12,13], where well-localized faults in both the inner and outer races of the bearing are diagnosed. These methods have demonstrated good results for cyclic or repetitive faults with well-defined frequency components; however, they are less effective for diffuse and non-cyclic faults, such as corrosion, where the signals are irregular and complex to characterize by means of specific or regular frequencies. For example, ref. [14] analyzed multiple condition parameters, including vibration, using wavelet and empirical mode decomposition for feature extraction, in order to diagnose cyclical faults in the outer raceway, such as holes of varying dimensions. FFT and statistical indicators such as skewness, kurtosis, standard deviation, Shannon entropy, and logarithmic energy have also been used to characterize the behavior of bearings under various damage conditions, demonstrating that these indicators can quantify fault severity [15,16]. Alternative methods based on stator current, acoustic analysis, and vibration signals have also been explored, showing that parameters such as root mean square (RMS) value and kurtosis are sensitive to the presence of defects [17,18,19]. Nevertheless, these signals can be susceptible to noise, since the changes induced by bearing defects tend to be subtle, making reliable diagnosis difficult [20,21]. Most of these studies focus on cyclic faults; therefore, frequency-domain or time–frequency methods tend to yield adequate results. For example, a fault due to material loss on a specific side of the outer bearing race will tend to cause vibrations each time the bearing balls pass over that area.

In contrast, corrosion damage in induction motor bearings, according to [6], tends to present problems along the raceways and does not usually exhibit cyclical patterns. In this regard, some papers [18,20] have proposed the analysis of stator current signals by means of spectral components. These papers focus on doubly fed induction generators used in wind turbines and in synchronous reluctance motors; however, induction motors are not analyzed or mentioned. In [18], electrical corrosion is generated in specific areas of the bearing, thus generating a localized fault, which is diagnosed by means of the modulation signal bispectrum. Regarding [20], bearing corrosion is diagnosed from stator current signals using the modulation signal bispectral detector, allowing the identification of amplitude and phase relationships associated with torque oscillations caused by corrosion, revealing characteristic spectral components even at incipient stages of damage. On the other hand, some papers have addressed the pitting damage in bearings of induction motors. This damage is characterized by small pits or craters in the rolling elements, caused by electrical corrosion. Corrosion in bearings, as mentioned previously, is usually studied through the analysis of localized pitting, which generates cyclic patterns associated with damage in specific areas of the bearing and can be evaluated using frequency- or time–frequency-domain techniques. For example, in [22], pitting in induction motor bearings is analyzed based on the electric bearing impedance, showing that this type of damage generates peaks in the impedance signal that can be used for diagnosis. Other works [23,24,25,26,27,28] also address pitting corrosion in electric motor bearings, focusing on localized faults and applying different methodologies based on time–frequency analysis of vibration or electrical signals. In [29], corrosion is analyzed using statistical indicators extracted from stator current signals in synchronous reluctance motors (different from induction motors, which are the most widely used worldwide), showing that electrical signals can also reflect damage associated with corrosion. However, all these approaches depend on repetitive fault patterns and are sensitive to noise and the variability of the corrosion process; moreover, many do not focus on induction machines. In general, the available technical literature mainly treats corrosion as localized pitting and does not rely on real industrial environments or practical scenarios, as explained in [6]. It is also worth highlighting that there are no methodologies that can diagnose induced corrosion due to humidity faults in induction motor bearings; therefore, comparisons of the proposed approach will be directly with works that analyze corrosion pitting.

In this regard, the use of advanced algorithms based on ML- and AI-based methodologies has further improved diagnostic performance. Some papers, such as [30,31,32], implemented advanced algorithms and data fusion techniques, combining electrical and vibration signals to enhance fault classification, increase robustness, and improve the separability between healthy and faulty bearing conditions. Other studies have compared multiple ML algorithms for bearing fault detection in IMs to determine which algorithm offers the highest accuracy. For example, ref. [33] evaluated vibration signals under different fault conditions present in the inner race, outer race, and ball defects, finding that SVM achieved 100% accuracy in detecting ball faults, while the k-nearest neighbors (KNN) algorithm had the best performance across the three fault types. In [34], thermographic images were processed using the two-dimensional discrete wavelet transform and principal component analysis (PCA) to extract relevant features to diagnose self-aligning bearings for rotating machinery, demonstrating that SVM outperformed linear discriminant analysis (LDA) and KNN. Other studies [35,36,37] have used artificial neural networks (ANNs) and SVM, optimized through genetic algorithms; both methods achieved effective classification, with SVM generally being superior. More recently, ensemble classifiers such as bagged trees and Gaussian process regression have also been employed, achieving high accuracy in detecting bearing degradation [38]. Additionally, recent studies have explored advanced sensing techniques beyond conventional frequency-based methods to improve bearing fault diagnosis. For instance, in [39,40], the use of acoustic emission signals is investigated to capture early-stage damage mechanisms associated with bearing degradation. These works highlight that acoustic emission is highly sensitive to micro-scale phenomena such as friction, impacts, and material loss, allowing the detection of faults that may not yet be clearly observable in vibration or current signals. The results emphasize the potential of acoustic emission as a complementary source of information for diagnosing complex bearing faults under realistic operating conditions.

Although several studies have compared machine learning and AI techniques for bearing fault diagnosis, there is still a lack of work exploring ensemble strategies, particularly bagging, and no combination of these techniques with stray magnetic flux analysis has been reported for diagnosing corrosion due to humidity. While frequency-based methods, as previously discussed, present certain limitations when dealing with complex and distributed damage, AI-based approaches have shown clear advantages by overcoming these drawbacks and enabling the study of fault mechanisms that are difficult to characterize using traditional techniques.

Therefore, the present work aims to develop a methodology capable of diagnosing corrosion due to humidity in IM bearings under various severity levels. This is achieved by using ensemble learning algorithms, such as bagging, and optimization algorithms to process both vibration and magnetic stray flux signals, thereby refining parameter optimization to improve accuracy.

## 2. Materials and Methods

This section describes the proposed methodology for diagnosing corrosion in IM bearings in detail, including the algorithms used, the justification for using both magnetic stray flux and vibration signals, and the subsequent experimentation.

### 2.1. A Characteristic Equation for Bearing Faults

In the analysis of faults in the bearings of IMs, it is essential to understand the dynamic interaction that exists between the rotor and the stator, as well as the effects that mechanical faults produce on electrical signals and vibration signals. As mentioned earlier, faults usually manifest as localized or specific defects that develop in certain contact regions, such as the inner race, the outer race, or the rolling elements, generating imperfections that appear as material losses or bulges. These imperfections cause periodic impacts at regular intervals related to the rotation frequency. The repetition of these impacts produces frequency patterns that are characterized through conventional techniques, such as FFT and other frequency-based methodologies.

**Figure 1:**
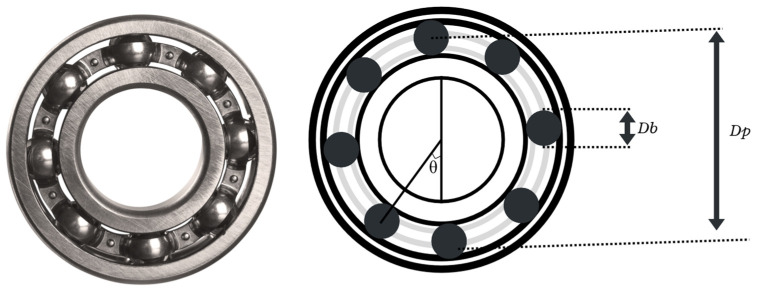
Bearing geometry and main parameters such as ball diameter, raceway diameter, and contact angle.

To identify these faults, characteristic equations are used to calculate the frequencies associated with different types of defects. These equations are derived from the geometry of the bearing, as can be seen in Figure 1, and from the movement of its components. These allow estimation of the frequency at which a ball passes over an imperfection located on the inner or outer race, as well as the frequencies related to defects in the rolling elements or the cage. These characteristic frequencies are quite important in vibration spectrum analysis, since they tend to produce peaks around those frequencies, allowing the identification of specific types of faults.

The development of these equations requires key parameters such as the ball diameter, pitch diameter, number of rolling elements, contact angle, and rotational speed of the shaft. These values are essential to calculate the most common fault frequencies in IM bearings, which are summarized in Table 1. The most typical defects include the inner race fault (BPFI), outer race fault (BPFO), rolling element fault (BSF), and cage fault (FTF). Equation (1) is used to determine inner and outer race fault frequencies, based on the concept of periodic impacts generated as the balls pass over a damaged area. Equation (2) is applied to faults in the rolling elements, which introduce additional modulations into the signals [41,42].

**Table 1:** Characteristic fault frequencies of ball bearings.

Fault	Equation	
Ball pass frequency of the outer race and inner race	$f_{rc}=\frac{N_{b}\times f_{s}}{2}\left[1\pm \frac{D_{b}}{D_{p}}\cos\left(\theta \right)\right]$	(1)
ball faults	$f_{cbf}=\left|f_{s}\pm n{\times f}_{rc}\right|$	(2)

where

○ $f_{cbf}$ is the component generated by bearing faults.
○ $f_{s}$ is the motor supply frequency.
○ n is an integer value (1, 2, 3…) determining fault frequency harmonics.
○ $f_{rc}$ are the characteristic fault frequencies for the inner and outer races.
○ $N_{b}$ is the number of bearing balls.
○ $D_{b}$ is the diameter of the bearing balls.
○ $D_{p}$ is the bearing pitch diameter.
○ θ is the contact angle between the ball and the race.

It is important to note that these equations are the basis of diagnostic methodologies based on frequency- or time–frequency-domain analysis. However, it should be emphasized that they allow the correlation of peaks around the rotation frequency with harmonics specific to localized faults, such as corrosion pits on the bearing balls, which, as mentioned earlier, represent a type of corrosion fault addressed in the literature. When the damage is extensive, such as corrosion due to humidity [6], the spectral patterns become less predictable, which considerably hinders fault diagnosis and requires other, more reliable types of analysis.

### 2.2. Humidity-Induced Bearing Corrosion

Corrosion can be triggered by a combination of one or more problems and is therefore one of the main reasons bearings are replaced in induction motors. If such a fault is not detected in time, it can lead to catastrophic damage, affecting other motor components and making it difficult to determine the root cause of the problem.

According to the SKF manual, corrosion can occur in two main ways: due to humidity or friction. One of the most noticeable consequences of this type of failure is the formation of oxidation and rust as a result of chemical reactions. In addition, corrosion pitting, flaking, and etching can also develop. It is quite common for ineffective seals to allow the ingress of moisture, water, or aggressive liquids into the bearings, leading to corrosion. Rust can also form if the liquid exceeds the bearing’s lubrication capacity and its ability to protect the steel surfaces. It is important to note that oxidation and corrosion are two distinct yet closely related processes. On the one hand, oxidation forms a protective oxide layer on the steel surface of the bearing when exposed to air; when water or humidity comes into contact with the steel, oxidation begins. According to the SKF manual [6], corrosion represents the main cause of premature bearing failures in motors used in the food and beverage industries, two of the most demanding sectors worldwide.

**Figure 2:**
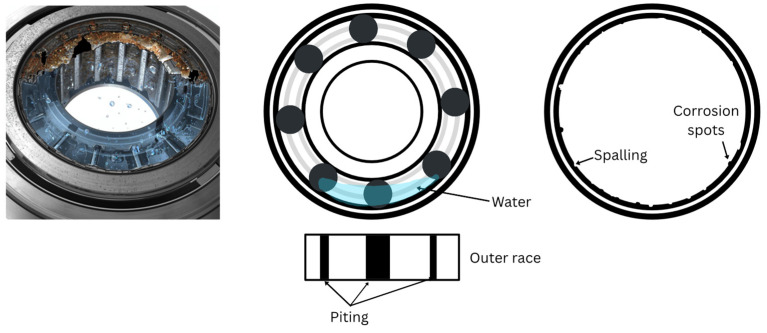
Deep corrosion (etching) on the outer race of the bearing, showing the formation of corrosion spots and spalling.

Corrosion develops from the oxidation process, causing the material to lose its original properties. Etching, on the other hand, refers to deep corrosion caused by the penetration of water, moisture, or aggressive liquids—such as acids—between the rolling element and the raceway. Over time, this leads to deep pitting and peeling, as shown in Figure 2 and as mentioned above.

Mechanical faults in IMs, such as eccentricities, misalignments, or bearing corrosion, can significantly influence rotor dynamics and, consequently, alter the magnetic field distribution within the air gap [43]. It has been reported that bearing defects may cause radial displacements that can be either periodic or non-periodic, depending on the location and type of damage. These displacements modify the air-gap length and, therefore, the magnetic permeance. Such variations lead to distortions in the magnetic stray flux, producing amplitude and phase modulations in the air-gap magnetic field. When a bearing fault occurs, a periodic displacement is induced, making the air-gap permeance term time-dependent and generating additional components in the magnetic flux density. This flux can be approximated as shown in Equations (3) and (4), where B(θ,t) represents the variations in the magnetic flux density, generating amplitude and phase modulation in the air-gap flux; $\Lambda_{0}$ represents the nominal air-gap permeance; $\Lambda_{1}$ represents the variation induced by the fault; and $f_{c}$ is the characteristic fault frequency. For localized defects, this modulation gives rise to sideband components around the fundamental frequency of the magnetic field, which are later transferred as sidebands in the current spectrum.

(3) $$B\left(\theta ,t\right)=F_{tot}\left(\theta ,t\right)\times \Lambda (\theta ,t)$$

(4) $$B\left(\theta ,t\right)=F_{tot}\left(\theta ,t\right)\left[\Lambda_{0}+\Lambda_{1}cos(2\pi f_{c}t)\right]$$

Thus, mechanical irregularities in the bearings of IMs can cause fluctuations in the magnetic coupling between the stator and rotor, leading to modulation of the air-gap flux. Consequently, these disturbances propagate beyond the main magnetic circuit, producing variations in the magnetic stray flux, which can be measured on the external surface of the motor. It is well known that the stray flux represents a fraction of the total magnetic field; therefore, any asymmetry or distortion in the air-gap flux is directly reflected in it. As a result, mechanical faults that initially appear as radial or angular displacements can modulate the distribution of the magnetic flux. These principles are fundamental for understanding how magnetic stray flux is affected by different mechanical bearing faults, including corrosion phenomena. However, corrosion, being a diffuse type of damage spread across the bearing surface, produces non-deterministic fault frequencies while still generating amplitude modulation. This type of behavior can be captured using time-domain-based techniques, as discussed in this work. Furthermore, the vibrations induced by corrosion in IM bearings, together with the variations in magnetic stray flux, form the basis for selecting these two physical quantities to train an ensemble learning model such as bagging.

### 2.3. Use of Statistical Indicators

Once the signals are acquired, the current, magnetic stray flux, and vibration signals are processed in the time domain to perform an accurate diagnosis, considering the difficulty of diagnosing faults through frequency-domain parameters. For this purpose, windowing and overlapping techniques can be applied, as they allow the original signal to be divided into smaller and overlapping segments, thus capturing more subtle features that could be lost when examining the signal globally; in other words, the signals are analyzed with greater sensitivity to changes. In the case of faults with diffuse patterns, such as corrosion due to humidity, windowing and overlapping make it possible to detect amplitude variations and extract dynamic features that would be difficult to analyze otherwise.

To characterize and analyze the dynamic behavior of the signals, both statistical and non-statistical indicators are used. Statistical indicators describe the behavior of a signal in terms of magnitude, variability, and dispersion; that is, they characterize it mathematically, providing information about its temporal distribution. Examples include the RMS, standard deviation, and kurtosis, which allow the detection of subtle changes in motor dynamics that may be associated with faults such as imbalance, misalignment, or corrosion. On the other hand, non-statistical indicators analyze more specific physical properties of the signals, such as energy, complexity, and others. The use of both types of indicators is important because it improves diagnostic capability, providing models such as ML or AI algorithms with a clearer understanding of the system’s dynamic behavior. By capturing not only differences in signal behavior but also in energy- or physics-based patterns, this approach allows for distinction between normal and faulty conditions. The parameters presented in Table 2 include metrics such as standard deviation, kurtosis, fifth moment, sixth moment, RMS, skewness, Teager–Kaiser energy, and sample entropy, as well as their mathematical expressions.

**Table 2:** Equation of statistical and non-statistical parameters.

Statistical and Non-Statistical Parameters	Equation	
Standard deviation	$\sigma =\sqrt{\frac{1}{N}\times \sum_{i=1}^{N}{\left(X_{i}-M\right)}^{2}}$	(5)
Kurtosis	$K=\frac{\sum {\left(X_{i}-M\right)}^{4}}{\sigma^{4}}$	(6)
Fifth moment	$FM=\frac{\sum {\left(X_{i}-M\right)}^{5}}{\sigma^{5}}$	(7)
Sixth moment	$M=\frac{\sum {\left(X_{i}-M\right)}^{6}}{\sigma^{6}}$	(8)
RMS	$RMS=\sqrt{\frac{1}{N}\times \sum_{i=1}^{N}{\left(x_{i}\right)}^{2}}$	(9)
Skewness	$A=\frac{\frac{1}{n}\sum_{i=1}^{N}{\left(X_{i}-M\right)}^{3}}{\sigma^{3}}$	(10)
Teager–Kaiser energy (tkeo)	$\gamma \left[x\left(t\right)\right]={\left[\dot{x\left(t\right)}\right]}^{2}-x\left(t\right)\ddot{x\left(t\right)}$	(11)
Sampling entropy (sampen)	$ES=-\sum_{i=0}^{N}p\left(x_{i}\right)\times \log_{2}\left(p(x_{i})\right)$	(12)

where

○ $X_{i}\;\mathrm{and}\;X(t)$ is the time-domain signal (current, vibration, and magnetic stray flux).
○ σ is the standard deviation.
○ M is the meaning of the signal (current, vibration, and magnetic stray flux).
○ N is the number of data points.
○ N(e) is the minimum number of squares required to cover the time series with squares of size e.

### 2.4. Analysis of Variance

The analysis of variance (ANOVA) is a useful tool to comprehend the behavior and differences between signals of the same type. In the case of IMs, this analysis is useful to determine which statistical parameters tend to show better performance for diagnosing corrosion faults. By evaluating the differences between current, magnetic stray flux, and vibration signals, it precisely measures the variability of each signal to determine whether observable differences exist between conditions, thus indicating whether such differences are statistically significant. By applying this mathematical method, it is possible to identify which signals show greater sensitivity to bearing corrosion and which cases exhibit similar behavior under different conditions, indicating a low diagnostic capability. This analysis can also be useful to understand which signals may have a greater ability to provide useful information for the subsequent application of other advanced analyses.

According to the reviewed literature, it has been determined that stator current signals tend to show very subtle changes in response to mechanical faults, which limits their effectiveness for reliable diagnosis [20,21]. Therefore, by comparing the results obtained through ANOVA for each signal, it is possible to quantitatively verify whether the current provides useful information or, as observed in previous studies, presents a low diagnostic capability. For this reason, this method not only helps to select the most reliable signals but also experimentally validates the observations made in the literature regarding the effectiveness of each signal, allowing for a more accurate analysis.

### 2.5. Support Vector Machine Classifier

**Figure 3:**
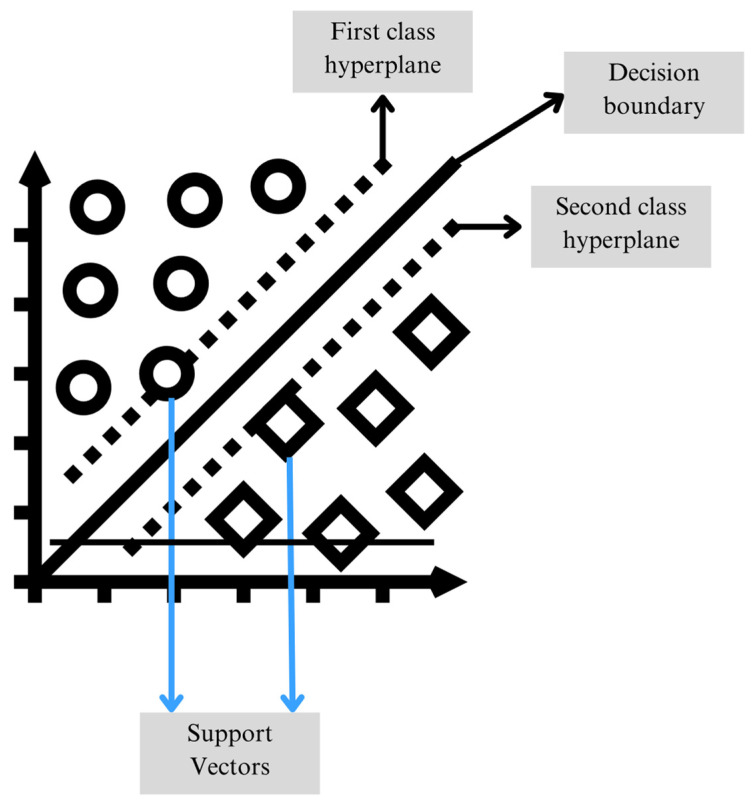
Illustration of the SVM classifier with the decision boundary, hyperplanes, and support vectors.

SVMs are machine learning tools used for classification, and their main objective is to find a separating hyperplane in a high-dimensional feature space that maximizes the margin between classes and minimizes classification error, as shown in Figure 3. To achieve this, SVMs map the input data into a higher-dimensional space where they can be separated by a hyperplane, generally performing class-by-class comparisons. If the data to be analyzed are linearly separable, the SVM optimizes the distance between two parallel hyperplanes that contain the closest points of each class, known as support vectors.

On the other hand, when the classes are not linearly separable, SVMs use kernel functions to transform the data into a higher-dimensional space where it is more likely to find a hyperplane that separates them effectively. This approach allows SVM to be a technique that provides greater robustness and effectiveness for fault detection and diagnosis problems, as it can handle large volumes of data and adapt to various configurations without requiring extensive tuning of hyperparameters [38].

### 2.6. Bagging Analysis

**Figure 4:**
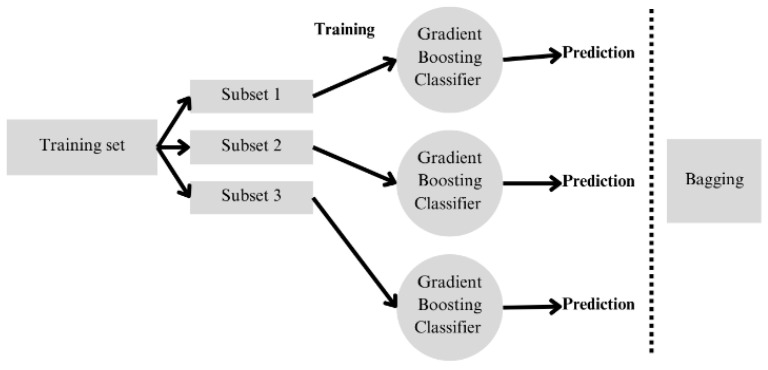
Schematic representation of the bagging ensemble classification method.

The ensemble learning algorithm technique, such as bagging, improves the accuracy of prediction and diagnostic models, since they are trained with different subsets of data (for this work, the base models will be SVMs) through bootstrap resampling; that is, for each of the obtained signals, by selecting the most significant indicators using a specific training set, various base classifiers are trained. This is very useful for avoiding overfitting (which occurs when models learn the specific noise from the training set), due to the use of different data samples and averaging of the results. To calculate this parameter, the relative overfitting rate (ROR) is used, where if the value tends to 0, the model does not present overfitting. Since each classifier provides a different response, majority voting is carried out to obtain a final classification on the test set, as shown in Figure 4.

### 2.7. Genetic Algorithm

Genetic algorithms are simulators of natural evolution used to solve optimization problems, such as selecting the best features or minimizing or maximizing variables. They generate populations of viable solutions, which are called individuals, and evaluate their quality, known as the fitness function. Then, selection, crossover, and mutation operations are applied to create new generations and thus prevent the obtained solutions from getting stuck in local solutions. Over time and through iterations, the fittest solutions are preserved, approaching the best viable solutions. Detailed information can be obtained in [44].

### 2.8. Sensor Instrumentation

**Figure 5:**
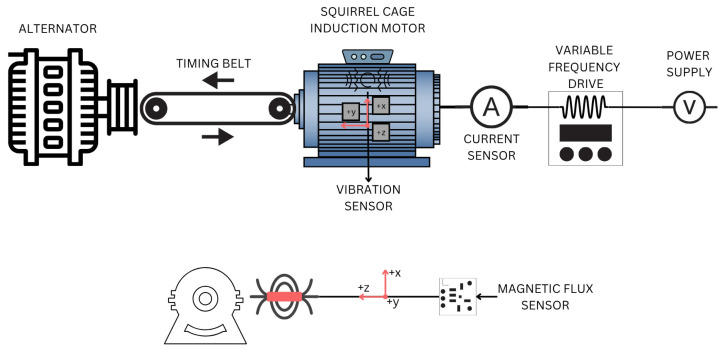
Sensor configuration and arrangement within the experimental setup.

In this study, various sensors (Figure 5) were used to acquire the signals necessary for analysis. A Fluke i200s clamp meter (Fluke Corporation, Everett, WA, USA), known for its accuracy and reliability, was used to measure current. Magnetic stray flux readings were taken with a BM1422AGMV three-axis magnetometer (ROHM Semiconductor, Kyoto, Japan), which provided measurements in all three directions. A triaxial vibration sensor, integrated into the data acquisition board used to acquire the magnetic flux, was also used. This board, based on FPGA technology, allows readings at a frequency of 1 kHz for both vibration and magnetic stray flux signals, which is useful for identifying highly detailed aspects of fault detection in induction motors. Furthermore, the sensors offer resolutions of 23 nA/LSB for current, 0.042 μT/LSB for magnetic stray flux, and 0.00479 m/s^2^/LSB for vibrations.

### 2.9. Proposed Methodology

**Figure 6:**
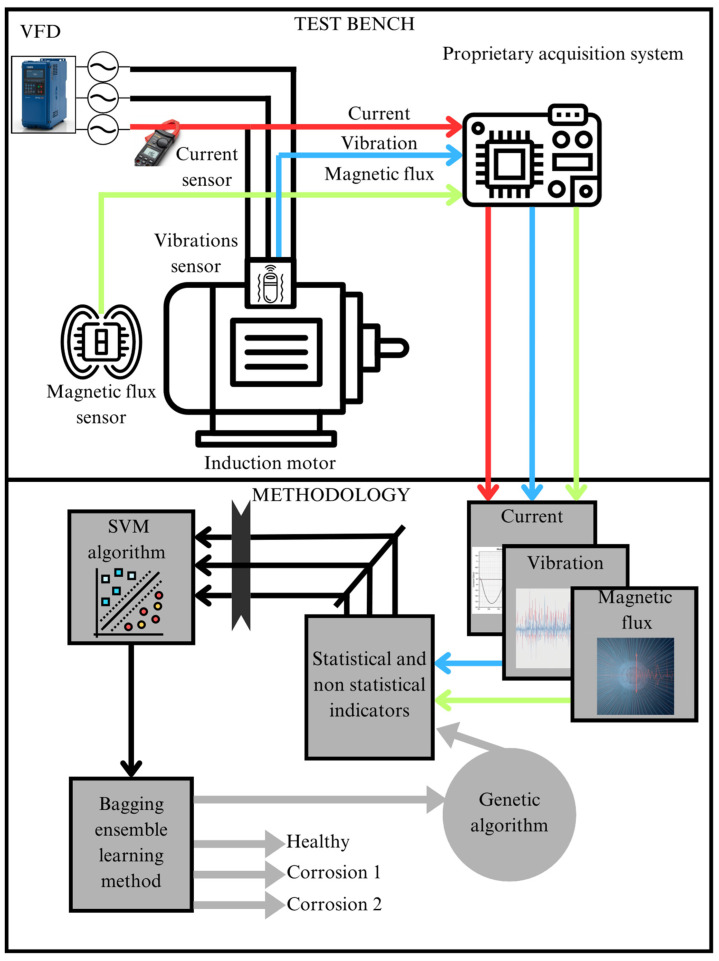
Proposed methodological flowchart.

The diagram in Figure 6 shows the two main components of the proposal: the test bench and the proposed methodology. The test bench contains the elements used during experimentation, such as equipment and tools to carry out the tests; it is mainly composed of a variable frequency drive (VFD) that allows the IM to operate at a frequency of 60 Hz. The motor is monitored by three main sensors: a magnetic flux sensor, a vibration sensor, and a current sensor. The signals from these sensors are captured through a proprietary data acquisition system. These time-domain signals represent the behavior of the motor under different conditions (healthy and with different levels of corrosion) and are used as inputs for the second stage of the process: the methodology. In this stage, the current, magnetic stray flux, and vibration signals are processed and analyzed using the statistical and non-statistical indicators described in Equations (5)–(12) to characterize the dynamic behavior of the signals according to the condition of the motor.

**Figure 7:**
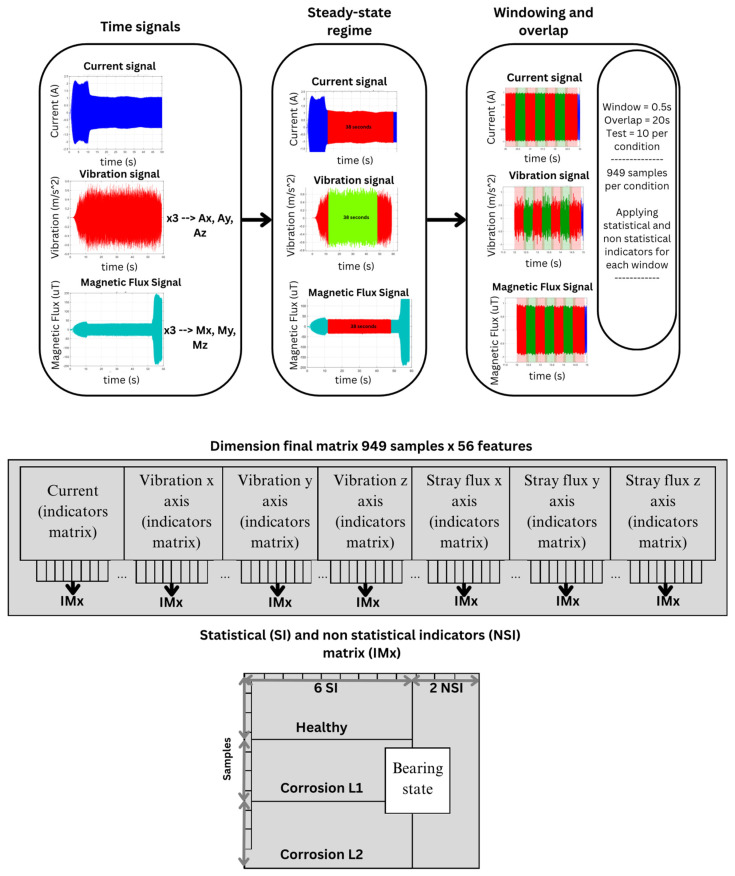
Final feature matrix.

Each of the features obtained through the statistical and non-statistical parameters feeds a classifier based on an ensemble learning algorithm, such as bagging, with an SVM as the base classifier. Finally, a genetic algorithm is implemented that works with a population of binary vectors, where each vector represents a potential solution to the feature selection problem, with a fitness function to minimize the bagging ensemble learning algorithm classification error rate. Each element of the vector corresponds to a feature, where a value of 1 indicates that the feature is selected, while 0 means it is not. The population size (20) defines the number of binary vectors (see Figure 7) in the population, and the number of generations (15) specifies how many iterations the algorithm will perform to improve the solutions. On the other hand, the mutation rate (0.1) determines the probability of modifying the selection of a feature (from 0 to 1 or vice versa) in an individual’s vector, which helps the algorithm explore new subsets of features without getting stuck in a local solution. The tournament size (3) is used during the parent selection process, where a random subset of individuals is chosen, and the one with the best fitness is selected for crossover. These hyperparameters are crucial for guiding the search for an optimal subset of features during the evolutionary process, in which the genetic algorithm optimizes both the feature selection and the random state hyperparameter of the bagging classifier. The values were adjusted through a trial-and-error procedure until the model performance stabilized and no further significant improvements were observed. In this context, the ROR metric was adopted as the main evaluation strategy to verify that the optimized model does not overfit and maintains consistent performance across the evaluated conditions, while the key parameters of the bagging ensemble learning algorithm were tuned to achieve improved and reliable diagnostic performance.

**Figure 8:**
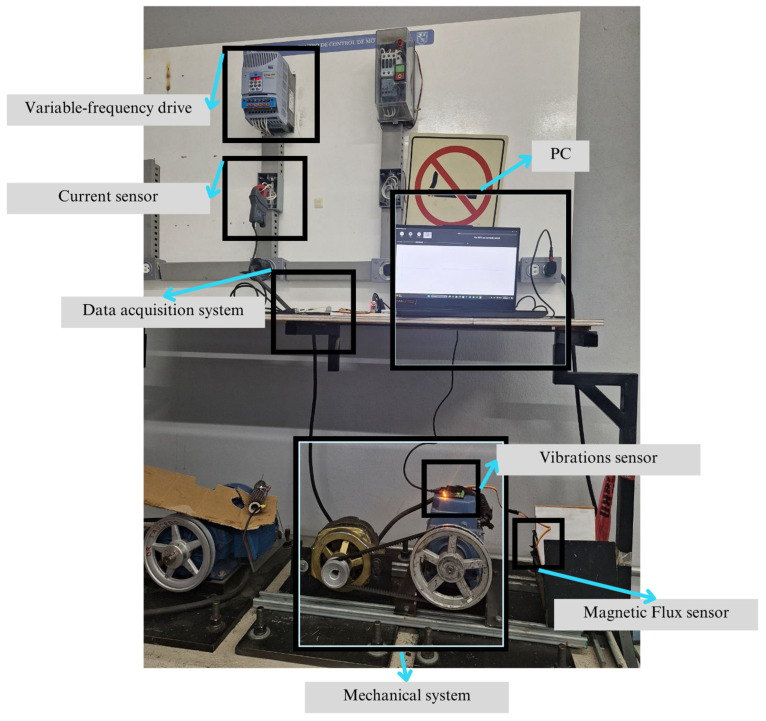
Test bench configuration.

Each operating condition (healthy state, corrosion level 1, and corrosion level 2) consists of 10 tests, each with a duration of 38 s (380,000 samples), and is normalized to the reference signal at values of −1 and 1, with the 10 tests for each condition carried out under the test bench shown in Figure 8. The signals are segmented using a windowing process with a fixed window length of 0.5 s and a 20% overlap. This procedure results in a total of 949 windows. Statistical and non-statistical indicators are applied to each window, as shown in Figure 7, generating a large set of statistical samples per condition and finally obtaining a 949 (samples) × 56 (features) matrix. Based on this, for each condition, the data was partitioned as follows: 70% of the samples were used for classifier training, and the remaining 30% for the testing phase. In this way, approximately 664 samples per condition were used for training and 285 samples per condition for evaluating the model’s performance. The model testing and training were performed on an ASUS TUF Gaming A15 laptop, which features an 8-core, 16-thread AMD Ryzen 7 4800H processor (Advanced Micro Devices, Inc., Santa Clara, CA, USA), 16 GB of 3200 MHz DDR4 RAM (Samsung Electronics, Suwon, South Korea), and an NVIDIA Ge-Force GTX 1660 Ti graphics card (NVIDIA Corporation, Santa Clara, CA, USA). A 512 GB NVMe SSD was used for storage, enabling efficient handling of large data volumes and significantly reducing loading and signal processing times.

### 2.10. Experimental Setup

Figure 8 shows the three-phase IM, which is coupled to an alternator by means of a toothed belt and pulleys, representing an approximate load of 30%, and operated at its nominal frequency of 60 Hz. A total of 10 tests were conducted for each bearing condition. A WEG SFW 08 frequency drive (WEG Industries, Jaraguá do Sul, Brazil) was used to control the operating conditions, allowing the motor input frequency to be adjusted.

The signals were recorded over a period of 48 s, of which 38 s corresponds to the steady-state operation of the IM. A proprietary 12-bit data acquisition board was used for vibration and magnetic stray flux measurements, with a sampling frequency of 1000 Hz. The flux sensor has a 14-bit resolution, and the vibration sensor has a 16-bit resolution.

**Figure 9:**
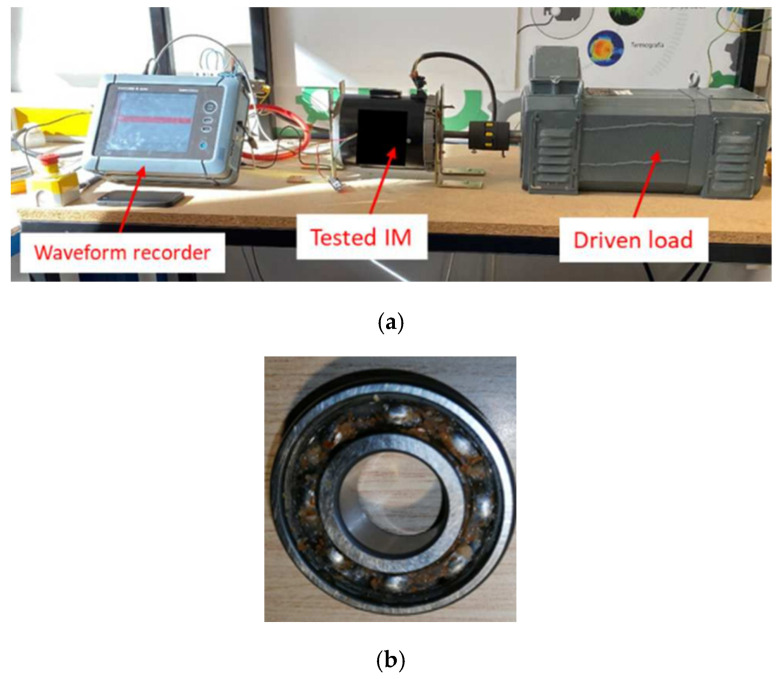
Second motor (**a**) test bench configuration and (**b**) corrosion due to humidity bearing.

For the validation of the proposed method, a second induction motor, different from the one used during the training phase, was employed. The main objective was to evaluate the generalizability of the proposed methodology’s model. Figure 9a shows the configuration of the motor mount used, which consists of the induction motor coupled to a drive load. Signals were taken for two different operating load conditions: 75% and nominal load (100%). Figure 9b shows the bearing with induced corrosion (different from those analyzed in the training), which constitutes the analyzed failure condition.

The motor used was a 1 HP three-phase induction motor with a nominal voltage of 460 V, a nominal current of 1.8 A, an operating frequency of 60 Hz, and a nominal speed of 1140 rpm. Some of these characteristics differ from those of the motor used during the algorithm training, allowing for the evaluation of the diagnostic system’s performance under different conditions and its generalizability. The signals acquired for this second motor include the same signals as in the training, i.e., magnetic stray flux and vibrations, which have dynamic and statistical characteristics comparable to those used during the training stage, although coming from a different physical system.

**Figure 10:**
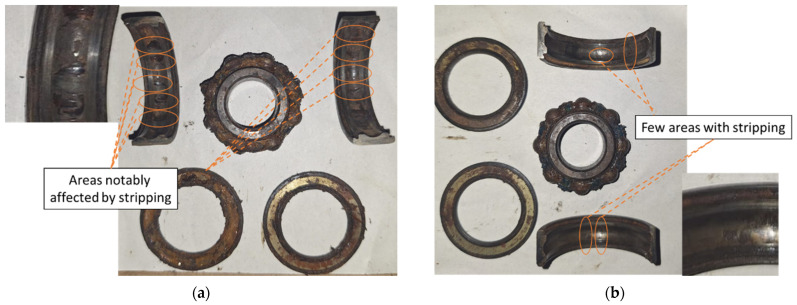
Corrosion (**a**) level 1 and (**b**) level 2 in the bearing, showing the damaged areas with etching and striping.

Figure 10 shows the condition of the bearings subjected to different corrosion severities. Figure 10a presents a bearing with the first severity level, referred to as corrosion level 1. Areas with initial signs of deterioration can be identified. In this case, the damage is quite mild and is characterized by the presence of corroded zones and material loss, as described in the SKF bearing manuals analyzed in the previous section. However, although the general structure of the bearing is not significantly affected, the presence of this type of corrosion indicates that structural damage already exists, perhaps not severe or extensive, but still present. The affected areas are limited, and the damage pattern is irregular, suggesting that the corrosion process has begun but has not yet reached a critical stage; nevertheless, initial surface striations can be observed.

On the other hand, Figure 10b shows a bearing with the second severity level, referred to as corrosion level 2, where the degree of damage is much greater, and the affected areas exhibit deep and extensive corrosion, with significant material loss in several contact zones. This damage pattern suggests a highly advanced corrosion state, in which wear and material loss have severely compromised the bearing’s condition. In the short term, this could lead to more serious problems, such as unexpected shutdowns or a reduction in the motor’s lifespan. Moreover, since the damage occurs across multiple non-localized areas, this reinforces the importance of employing advanced techniques beyond traditional frequency-domain transformations, which are useful for detecting fault patterns but may not be sufficient under such complex conditions.

## 3. Results

**Figure 11:**
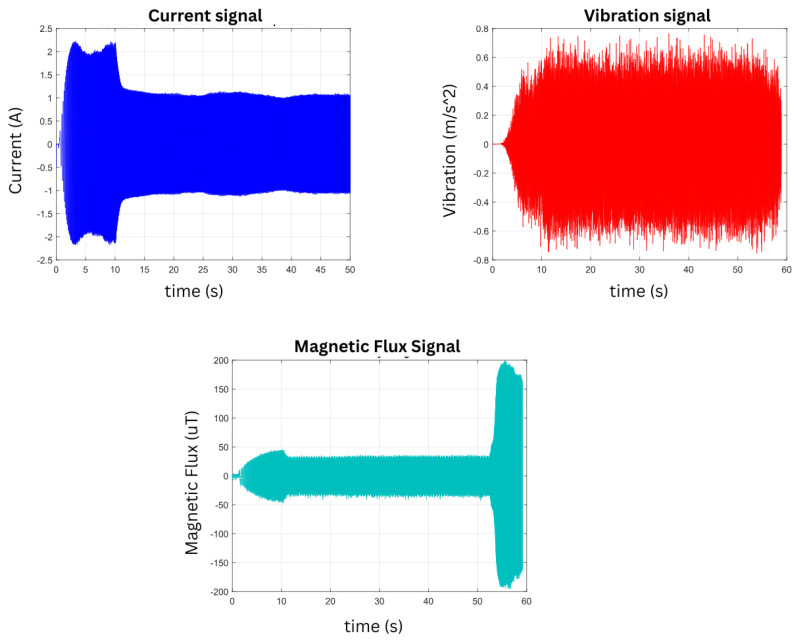
Time-domain representation of the current, vibration, and magnetic stray flux signals in the Healthy state.

Figure 11 shows the signals obtained from the IM under healthy operating conditions and two levels of corrosion, illustrating the time-domain response of the current, vibration, and magnetic stray flux signals. The current signal is shown first, exhibiting a stable behavior once the transient state has passed (approximately 10 s after the beginning of the test). The vibration signal is also presented, showing a noticeably more irregular behavior; unlike the current signal, it does not reach a clear steady state. Finally, the magnetic stray flux signal is presented, which shows a behavior similar to that of the current, with an initial transient phase followed by a well-defined steady state about 10 s after the start of the test. The signal exhibits a stable frequency and constant amplitude, indicating that the magnetic flux generated by the motor maintains a stable configuration after the initial adjustment period, reflecting proper operation of the electromagnetic system. These three signals are ready to be analyzed using advanced methods, since it would be difficult to diagnose faults or issues in the motor through simple visual inspection.

**Figure 12:**
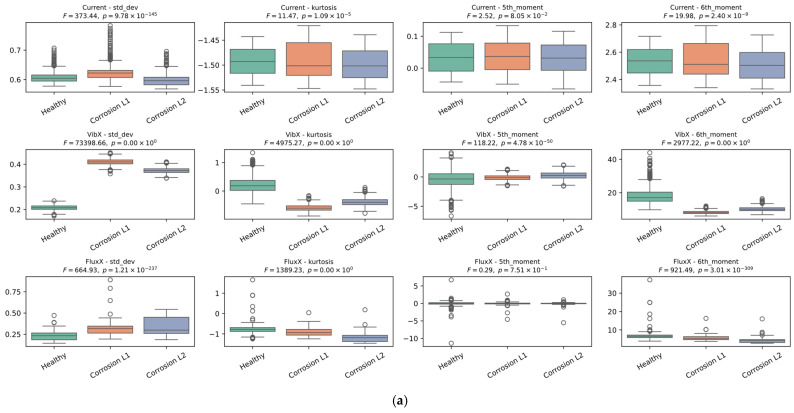

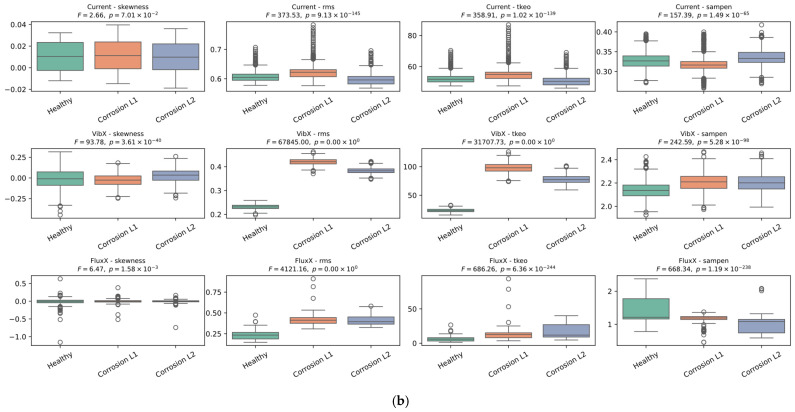
Results of the ANOVA performed on the three signals across the eight calculated statistical features. (**a**) Standard deviation, kurtosis, 5th moment, 6th moment. (**b**) Skewness, RMS, Teager–Kaiser energy (tkeo), sampling entropy (sampen).

Figure 12 shows the results obtained from the ANOVA analysis corresponding to each signal (current, magnetic stray flux, and vibrations) evaluated through the eight statistical indicators presented and characterized in Equations (5)–(12). The x-, y-, and z-axis locations of each sensor during experimentation can be observed in Figure 3. It is clearly observed that, for the current signal, the ANOVA results show a high degree of overlap between the operating and fault conditions, which agrees with the reviewed literature. This shows that the stator current tends to have subtle changes in response to bearing failures, which limits its diagnostic capability. On the other hand, for the vibration x-axis, it can be observed that the standard deviation indicator shows a more marked separation between the classes; this behavior is generally maintained for the kurtosis, sixth moment, RMS, and Teager–Kaiser energy indicators, which supports that the vibrations on this axis clearly reflect the variations associated with the corrosion fault in the bearings. For the magnetic stray flux signal, an increasing or decreasing behavior is observed in the distribution of the ANOVA graphs. Although the separation between classes is not as marked, this trend is quite important since it indicates that this signal is also useful for diagnosing the fault, providing relevant information. In this case, the RMS, standard deviation, Teager–Kaiser energy, and sample entropy indicators showed a more evident separation between the fault levels and the healthy state. Consequently, when applying machine learning algorithms such as SVM and bagging, the results are expected to be more reliable and accurate for the magnetic stray flux and vibration signals, which highlights the relevance of using the genetic algorithm to optimize the selection of the best combinations of features.

Given the best combination of statistical and non-statistical indicators obtained through the optimization process with the genetic algorithm, it was found that the best indicators for the vibration signal on the x-axis were standard deviation, skewness, and Teager–Kaiser energy; for the y-axis, sample entropy, RMS, and skewness were selected; and finally, for the z-axis, standard deviation, skewness, and RMS were selected. On the other hand, for the magnetic stray flux in the x-axis, standard deviation, kurtosis, fifth moment, and skewness were selected; for the y-axis, fifth moment, skewness, Teager–Kaiser, and sample entropy were selected; and for the z-axis, standard deviation, kurtosis, fifth moment, skewness, and RMS were selected. These indicators are consistent with those that showed better performance in the ANOVA analysis; however, the use of the genetic algorithm optimizes this selection.

The indicators selected by the genetic algorithm can be physically interpreted, since they capture changes introduced by corrosion in the dynamic and electromagnetic behavior of the system. Additionally, these indicators are influenced by variations in the air gap caused by bearing deformations due to corrosion, which alter the rotor–stator magnetic coupling, as explained in Section 2.3. Thus, the standard deviation and RMS can reflect variations in the energy and amplitude of the signals, which may be caused by surface irregularities and material loss, features that are strongly present in vibration signals. Skewness, kurtosis, and higher-order moments are tools that can identify changes in the signal distribution associated with corrosion. Teager–Kaiser energy highlights phenomena related to friction and the contact between the bearing balls and corrosion pits produced by induced corrosion due to humidity (non-localized). Sample entropy can show an increase in the complexity and irregularities of the signals as corrosion becomes more evident. For all these reasons, the genetic algorithm tends to favor the selection of these indicators, providing better separability between classes and maximizing robustness.

Subsequently, analysis and classification were performed using bagging with an SVM classifier as the base. This analysis was carried out on the training set, obtained by dividing the data into 70% for training and 30% for testing, which allowed for an objective evaluation of the model and thus avoided overfitting. The genetic algorithm also helped in selecting the best bagging indicators.

The analysis achieved an accuracy higher than 99%, reflecting the effectiveness of the method used. The combination of the genetic algorithm and bagging with SVM maximized the ability to discriminate between the healthy bearing condition and the two corrosion levels, demonstrating the robustness of the model when dealing with previously unseen data.

**Figure 13:**
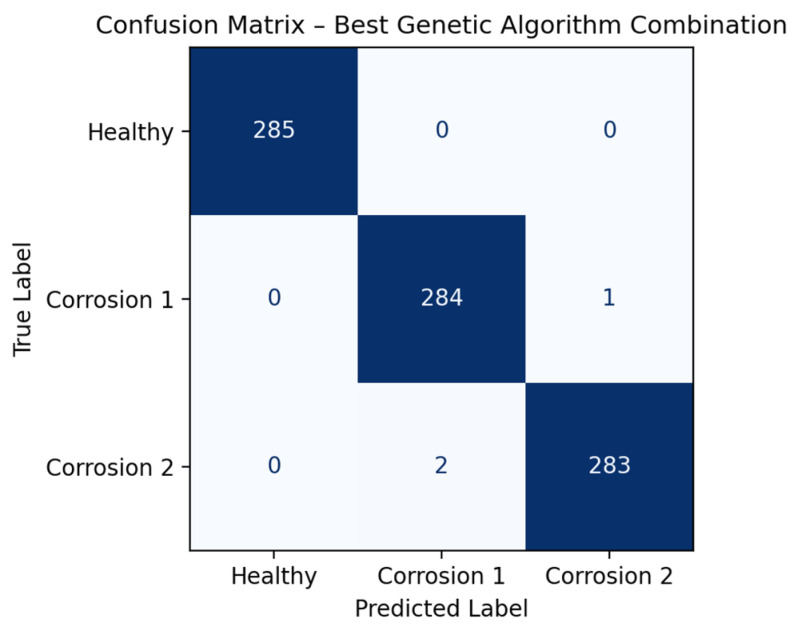
Confusion matrix classification results obtained after applying the bagging ensemble method.

Finally, Figure 13 presents the confusion matrix obtained from the bagging model, which achieved an accuracy close to 100%. Only one case of bearing with corrosion level 1 was misclassified as corrosion level 2, and two cases of bearings with corrosion level 2 were misclassified as level 1, highlighting the effectiveness of the approach in managing data variability.

A classification report was also obtained, where the healthy condition was correctly identified in all cases, achieving precision, recall, and F1-score values of 100%, which clearly demonstrates correct separation with respect to the classes focused on corrosion levels. For corrosion level 1, a precision of 98.95%, a recall of 99.65%, and an F1-score of 99.30% were obtained, while for corrosion level 2, a precision of 99.65%, a recall of 98.95%, and an F1-score of 99.30% were achieved; this is due to the fact that they present similar physical characteristics, as they correspond to different degrees of the same phenomenon, which explains the slight confusion observed between both classes. The high F1-score values confirm that the model is able to effectively capture the real progression of corrosion. These results show that the classes associated with bearing damage due to corrosion were correctly classified in almost the entire test set, demonstrating a high generalization capability of the model.

## 4. Discussion

The analysis of variance has proved to be fundamental for analyzing the signals that provide the most information for diagnosing corrosion faults in bearings. As mentioned earlier, the literature has documented that current signals present limitations for bearing fault diagnosis due to the low level of disturbances generated in the frequency spectrum and the inherent noise of industrial environments. The results presented here confirm this observation, since in the ANOVA analysis, both the statistical and non-statistical indicators failed to distinguish between fault states and the healthy state. This phenomenon is not observed in the magnetic stray flux or vibration signals, which were successfully differentiated through some of the statistical and non-statistical indicators, for example, for vibrations in the x-axis, the indicators of standard deviation, kurtosis, sixth moment, RMS, and Teager–Kaiser energy; and for magnetic stray flux, the indicators of RMS, standard deviation, Teager–Kaiser energy, and sample entropy. This behavior is consistent with previous studies indicating that corrosion affects the bearing air gap, modulating the magnetic stray flux and generating detectable patterns, while vibrations produced in the bearing regions with corrosion pitting generate motion each time a ball passes through these areas, thus modulating the signals.

Table 3 presents the comparative results of the approach using bagging combined with SVM versus SVM alone, evaluating both accuracy and the ROR for different types of signals: current alone, magnetic stray flux alone, vibrations alone, and the combination of magnetic stray flux and vibrations. It is clearly observed that the combination of magnetic stray flux and vibrations, together with bagging and SVM, provides the highest accuracy and the lowest overfitting, reaching an ROR of only 1.84%. This result is significant because it not only improves the predictive capability of the model but also ensures robustness and reliability. In contrast, methodologies that do not use bagging show higher overfitting rates, reaching up to 8% in the case of SVM alone, which could result in unreliable diagnoses in industrial environments where operating conditions are highly variable, potentially causing the training set to overfit and capture noise.

Model robustness is reflected not only in accuracy but also in the ability to generalize without overfitting, which is crucial for real industrial applications. As mentioned earlier, a system with high accuracy but elevated overfitting could generate false positives or negatives, affect maintenance planning, and increase costs associated with unexpected downtime. The integration of magnetic stray flux and vibrations captures both the electromagnetic effects of corrosion and the mechanical response of the bearing, providing a more comprehensive diagnosis.

Table 4 compares the proposed methodology with the approaches described in the literature. Most existing studies focus on cyclic or localized faults, generally related to corrosion pits on bearing balls, or apply their methods to motors other than the induction motors most widely used in the global industry. In contrast, the proposed approach follows the guidelines of the SKF manuals [6], which allows faults to be characterized as they occur in industrial practice, manifesting as material loss, pitting, spalling, or corrosion spots. This ensures that the diagnosis is representative of real industrial conditions and not only experimental scenarios. Among the reviewed works, the only study that can be considered comparable is [29], which addresses corrosion through electrical signals, but is applied to synchronous reluctance motors, a type of machine that is less common in industrial applications. In addition, ref. [18] presents a related approach focused on corrosion in bearings of doubly fed induction generators used in wind turbines. The remaining studies mainly analyze localized corrosion pits or pitting faults [14,22,23,24,25,26,27,28], which, although not fully comparable with the behavior of corrosion in real industrial environments, represent the most studied type of fault in the literature.

Although a localized fault can be diagnosed through frequency analysis, these methods have limited usefulness in the present work due to the complexity of real bearing behavior under corrosion-induced faults. The proposed methodology, in contrast, captures the interaction between different signals and their effects on the entire system, providing a robust and comprehensive diagnosis. Although frequency-based approaches are effective for identifying isolated pitting faults, they do not fully represent the distributed and evolving nature of corrosion observed in industrial practice. The achieved accuracy of 99.67%, in addition to the low ROR, demonstrates the strong capability to evaluate faults according to their real manifestation, with minimal need for generalization, highlighting the proposed methodology as a useful tool. This ensures that the proposed approach is not only accurate but also reliable and safe for implementation in industrial environments.

Once the classification model is trained, it is tested using data from a different motor operating under conditions and characteristics not included in the training set. During this stage, the model is not retrained or adjusted; instead, the external motor signals are processed using the same steps to project them onto the original feature space, allowing the evaluation of the model’s ability to identify damage patterns independent of the specific motor. Since the external data do not have ground-truth labels, the evaluation is not based on traditional metrics, and the results are instead analyzed through the distribution of inferred classes and their physical consistency. To support this analysis, the t-SNE technique is used solely as a visualization tool, projecting both training and external data into a reduced-dimensional space to observe whether the samples cluster according to the learned corrosion classes.

Thus, the previously trained classification model was evaluated using signals from the second motor, which exhibits bearing corrosion under two load conditions: 75% and 100% (nominal load). It is important to note that during this process, the model was neither retrained nor adjusted to the data from the second motor. Therefore, the classifier parameters and the feature selection obtained through the genetic algorithm remain unchanged. Inference was performed, which shows that 100% of the samples analyzed were classified as having level 2 corrosion. No data was classified as level 1 corrosion or as healthy. The accurate identification of corrosion level 2 under different load levels suggests that the model is quite capable of capturing damage-related characteristics, beyond the variations induced by the motor’s operating conditions. This enhances robustness to load variations, a critical aspect in real-world industrial applications where motors typically operate under diverse and varied conditions.

**Figure 14:**
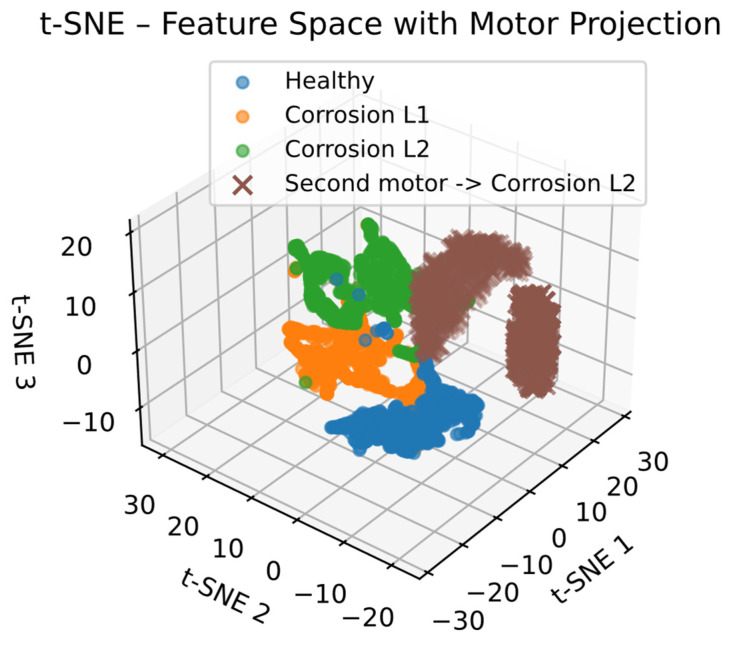
T-SNE based on feature space with motor projection.

With the purpose of clearly visualizing the behavior and distribution of the samples obtained with the proposed methodology, a projection of the different extracted indicators onto a 3D map was carried out for the various study cases using the t-SNE technique. Furthermore, the projection of characteristics using the t-SNE technique, shown in Figure 14, reveals that the samples corresponding to the signals from the second motor clearly cluster within the region belonging to corrosion level 2, as defined by the training test data. This grouping not only supports the numerical results obtained from the inference but also suggests that they are actually being classified within the same space assigned to corrosion level 2. Finally, the fact that the model was able to correctly generalize to a different machine operating under different conditions and without retraining shows the capabilities of the proposed method for application in scenarios where data availability and labels for each specific machine are often limited.

It is important to note that for future work, it would be useful to evaluate the proposed methodology under a greater number of operating conditions typical of real industrial environments, with the main objective of continuing to analyze the robustness of the model and its generalization capability. Likewise, the application of the approach to machines with different power ratings, greater load variations, or longer operating periods would allow the study of its behavior under more complex corrosion scenarios.

## 5. Conclusions

This work addressed the problem of diagnosing corrosion in induction motor bearings through the implementation of advanced classification and optimization techniques, from which the following conclusions can be drawn.

It is evident that the direct analysis of data through traditional statistical techniques, such as ANOVA, is insufficient to correctly separate the classes in the problem. However, these techniques provide interpretive support to verify and confirm the literature findings regarding the limited diagnostic capability of the current signal for bearing faults. In addition, some statistical parameters of both the stray magnetic flux and vibrations, evaluated through ANOVA, show separation among the three classes, which suggests that these two signals provide the most relevant information for subsequent analyses, such as bagging. This also confirms the findings in the literature, which indicate that bearing problems cause air-gap displacement, resulting in the modulation of stray magnetic flux signals, validating the common use of the vibration signals in combination with other methodologies to diagnose punctual faults.

For non-punctual faults, such as corrosion caused by humidity, advanced processing and classification techniques are necessary. In this study, a model based on the combination of bagging and support vector machines (SVMs) was developed, optimized through genetic algorithms. The use of bagging and SVM contributed to reducing variance and improving the classification capability of the model. The application of the genetic algorithm optimized feature selection, improving the accuracy of the model and reducing its complexity, as well as adjusting the main parameters of the bagging ensemble learning algorithm.

In addition, a comparative analysis with other methodologies demonstrated that the proposed model outperforms conventional approaches described in the literature. It was observed that the exclusive use of SVM resulted in lower accuracy compared to the combined bagging strategy, which confirms that the integration of dimensionality reduction and ensemble techniques significantly improves the model’s performance. The implementation of bagging helped reduce the overfitting rate, thus improving the generalization capability of the model. By training multiple classifiers on data subsets and combining their predictions, bagging decreased variance and reduced the impact of specific patterns from the training set, achieving more stable performance on test data.

Finally, the results validate the effectiveness of the implemented model for diagnosing corrosion in induction motor bearings under real operating conditions, as documented in the SKF manual, instead of addressing only isolated faults, as reported in the literature. The combination of ensemble and classification techniques allowed the development of a robust system with high generalization capability, suitable for industrial applications and predictive maintenance.

The results obtained using data from the external motor confirm that the proposed methodology maintains consistent performance even when applied to a different machine operating under distinct conditions and without retraining. The correct assignment of the samples to the regions corresponding to the previously learned corrosion levels indicates that the model captures general physical patterns of the phenomenon rather than characteristics specific to a single motor, supporting its generalization capability and its suitability for application in real industrial environments.

## Figures and Tables

**Table 3:** Comparative results of the bagging–SVM model versus conventional SVM using individual and combined signals (current, magnetic stray flux, and vibration).

Signal	Bagging + SVM		SVM	
	Accuracy (%)	ROR (%)	Accuracy (%)	ROR (%)
Current	53.35	0.64	44.04	5.65
Vibration	97.79	3.59%	96.32	3.97
Magnetic Stray Flux	96.84	6.02	96.54	6.82
Magnetic Stray Flux + Vibration	99.67	1.84	98.35	4.22

**Table 4:** Comparison of the proposed methodology with literature-reported approaches for bearing corrosion diagnosis.

Used Methodology	Accuracy (%)	Condition Type	Highlights
Proposed Methodology	99.67	Corrosion due to humidity	Corrosion according to the SKF manual
[29]	92	Corrosion	Corrosion in synchronous reluctance motor bearings
[14,22,23,24,25,26,27,28]	>88	Localized fault	Pit corrosion in balls
[18]	>90	Corrosion due to humidity	Corrosion in doubly fed induction generator bearings in wind turbines

## Data Availability

The data presented in this study are available on request from the corresponding author. The data is not publicly available due to other research works in progress using same data.

## Abbreviations

The following abbreviations are used in this manuscript:

IM	Induction Motor
ANOVA	Analysis of Variance
FFT	Fast Fourier Transform
AI	Artificial Intelligence
ML	Machine Learning
SVM	Support Vector Machine
KNN	K-Nearest Neighbors
PCA	Principal Component Analysis
LDA	Linear Discriminant Analysis
ANN	Artificial Neural Networks
RMS	Root Mean Square
BPFI	Inner Race Fault
BPFO	Outer Race Fault
BSF	Rolling Element Fault
FTF	Cage Fault
RBF	Radial Basis Function
VFD	Variable Frequency Drive
ROR	Relative Overfitting Rate
